# Supplementary material for: Pyruvate Dehydrogenase Kinase 1 inhibition mediated oxidative phosphorylation enhancement in cartilage promotes osteoarthritis progression
Source: BMC Musculoskelet Disord. 2023 Jul 20;24:597. doi: 10.1186/s12891-023-06585-6 (PMC10357736; doi:10.1186/s12891-023-06585-6)
Supplement: Supplementary file 1 — Additional file 1. [file 12891_2023_6585_MOESM1_ESM.zip › KEGG.Gsea.1653622432464/KEGG_INOSITOL_PHOSPHATE_METABOLISM.html]

Details for gene set KEGG\_INOSITOL\_PHOSPHATE\_METABOLISM[GSEA]

|  || Dataset | 55457\_collapsed\_to\_symbols.cls.cls #OA\_versus\_normal.cls.cls #OA\_versus\_normal\_repos |
| Phenotype | cls.cls#OA\_versus\_normal\_repos |
| Upregulated in class | normal |
| GeneSet | KEGG\_INOSITOL\_PHOSPHATE\_METABOLISM |
| Enrichment Score (ES) | -0.24858613 |
| Normalized Enrichment Score (NES) | -0.9591613 |
| Nominal p-value | 0.5295082 |
| FDR q-value | 0.7274416 |
| FWER p-Value | 1.0 |
Table: GSEA Results Summary

  

Fig 1: Enrichment plot: KEGG\_INOSITOL\_PHOSPHATE\_METABOLISM      
 Profile of the Running ES Score & Positions of GeneSet Members on the Rank Ordered List

  

| SYMBOL | TITLE | RANK IN GENE LIST | RANK METRIC SCORE | RUNNING ES | CORE ENRICHMENT || 1 | PLCG2 | phospholipase C gamma 2 [Source:HGNC Symbol;Acc:HGNC:9066] | 725 | 0.588 | -0.0100 | No |
| 2 | PIP4K2C | phosphatidylinositol-5-phosphate 4-kinase type 2 gamma [Source:HGNC Symbol;Acc:HGNC:23786] | 939 | 0.530 | 0.0152 | No |
| 3 | PIP5K1B | phosphatidylinositol-4-phosphate 5-kinase type 1 beta [Source:HGNC Symbol;Acc:HGNC:8995] | 1291 | 0.459 | 0.0242 | No |
| 4 | PLCE1 | phospholipase C epsilon 1 [Source:HGNC Symbol;Acc:HGNC:17175] | 1505 | 0.428 | 0.0414 | No |
| 5 | CDIPT | CDP-diacylglycerol--inositol 3-phosphatidyltransferase [Source:HGNC Symbol;Acc:HGNC:1769] | 1586 | 0.416 | 0.0680 | No |
| 6 | PIK3CB | "phosphatidylinositol-4,5-bisphosphate 3-kinase catalytic subunit beta [Source:HGNC Symbol;Acc:HGNC:8976]" | 2186 | 0.340 | 0.0482 | No |
| 7 | PLCB4 | phospholipase C beta 4 [Source:HGNC Symbol;Acc:HGNC:9059] | 2217 | 0.337 | 0.0724 | No |
| 8 | PLCD1 | phospholipase C delta 1 [Source:HGNC Symbol;Acc:HGNC:9060] | 2298 | 0.327 | 0.0920 | No |
| 9 | PIP4K2B | phosphatidylinositol-5-phosphate 4-kinase type 2 beta [Source:HGNC Symbol;Acc:HGNC:8998] | 3416 | 0.210 | 0.0216 | No |
| 10 | ITPKB | inositol-trisphosphate 3-kinase B [Source:HGNC Symbol;Acc:HGNC:6179] | 3467 | 0.205 | 0.0340 | No |
| 11 | PLCB1 | phospholipase C beta 1 [Source:HGNC Symbol;Acc:HGNC:15917] | 3724 | 0.183 | 0.0285 | No |
| 12 | TPI1 | triosephosphate isomerase 1 [Source:HGNC Symbol;Acc:HGNC:12009] | 4059 | 0.150 | 0.0144 | No |
| 13 | PIP4K2A | phosphatidylinositol-5-phosphate 4-kinase type 2 alpha [Source:HGNC Symbol;Acc:HGNC:8997] | 4400 | 0.121 | -0.0026 | No |
| 14 | PI4KB | phosphatidylinositol 4-kinase beta [Source:HGNC Symbol;Acc:HGNC:8984] | 4485 | 0.114 | -0.0001 | No |
| 15 | INPP4A | inositol polyphosphate-4-phosphatase type I A [Source:HGNC Symbol;Acc:HGNC:6074] | 5274 | 0.050 | -0.0575 | No |
| 16 | ALDH6A1 | aldehyde dehydrogenase 6 family member A1 [Source:HGNC Symbol;Acc:HGNC:7179] | 5560 | 0.028 | -0.0774 | No |
| 17 | ISYNA1 | inositol-3-phosphate synthase 1 [Source:HGNC Symbol;Acc:HGNC:29821] | 5973 | -0.003 | -0.1093 | No |
| 18 | INPP5J | inositol polyphosphate-5-phosphatase J [Source:HGNC Symbol;Acc:HGNC:8956] | 6009 | -0.005 | -0.1116 | No |
| 19 | MINPP1 | multiple inositol-polyphosphate phosphatase 1 [Source:HGNC Symbol;Acc:HGNC:7102] | 6055 | -0.009 | -0.1144 | No |
| 20 | ITPK1 | inositol-tetrakisphosphate 1-kinase [Source:HGNC Symbol;Acc:HGNC:6177] | 6095 | -0.012 | -0.1165 | No |
| 21 | INPP4B | inositol polyphosphate-4-phosphatase type II B [Source:HGNC Symbol;Acc:HGNC:6075] | 6284 | -0.026 | -0.1291 | No |
| 22 | SYNJ2 | synaptojanin 2 [Source:HGNC Symbol;Acc:HGNC:11504] | 6358 | -0.032 | -0.1322 | No |
| 23 | PIP5K1C | phosphatidylinositol-4-phosphate 5-kinase type 1 gamma [Source:HGNC Symbol;Acc:HGNC:8996] | 6601 | -0.050 | -0.1471 | No |
| 24 | PIK3C2B | phosphatidylinositol-4-phosphate 3-kinase catalytic subunit type 2 beta [Source:HGNC Symbol;Acc:HGNC:8972] | 6872 | -0.069 | -0.1627 | No |
| 25 | PLCB2 | phospholipase C beta 2 [Source:HGNC Symbol;Acc:HGNC:9055] | 7052 | -0.084 | -0.1700 | No |
| 26 | PLCB3 | phospholipase C beta 3 [Source:HGNC Symbol;Acc:HGNC:9056] | 7118 | -0.090 | -0.1680 | No |
| 27 | OCRL | OCRL inositol polyphosphate-5-phosphatase [Source:HGNC Symbol;Acc:HGNC:8108] | 7319 | -0.103 | -0.1754 | No |
| 28 | IMPA1 | inositol monophosphatase 1 [Source:HGNC Symbol;Acc:HGNC:6050] | 7332 | -0.105 | -0.1681 | No |
| 29 | PI4KA | phosphatidylinositol 4-kinase alpha [Source:HGNC Symbol;Acc:HGNC:8983] | 7397 | -0.110 | -0.1643 | No |
| 30 | PIK3CD | "phosphatidylinositol-4,5-bisphosphate 3-kinase catalytic subunit delta [Source:HGNC Symbol;Acc:HGNC:8977]" | 7405 | -0.111 | -0.1561 | No |
| 31 | PIK3CA | "phosphatidylinositol-4,5-bisphosphate 3-kinase catalytic subunit alpha [Source:HGNC Symbol;Acc:HGNC:8975]" | 7733 | -0.136 | -0.1708 | No |
| 32 | PIK3C2G | phosphatidylinositol-4-phosphate 3-kinase catalytic subunit type 2 gamma [Source:HGNC Symbol;Acc:HGNC:8973] | 7869 | -0.146 | -0.1698 | No |
| 33 | PIK3CG | "phosphatidylinositol-4,5-bisphosphate 3-kinase catalytic subunit gamma [Source:HGNC Symbol;Acc:HGNC:8978]" | 7953 | -0.152 | -0.1643 | No |
| 34 | INPP5K | inositol polyphosphate-5-phosphatase K [Source:HGNC Symbol;Acc:HGNC:33882] | 8103 | -0.163 | -0.1630 | No |
| 35 | PIK3C3 | phosphatidylinositol 3-kinase catalytic subunit type 3 [Source:HGNC Symbol;Acc:HGNC:8974] | 8141 | -0.166 | -0.1528 | No |
| 36 | INPP5A | inositol polyphosphate-5-phosphatase A [Source:HGNC Symbol;Acc:HGNC:6076] | 8813 | -0.218 | -0.1878 | No |
| 37 | PIK3C2A | phosphatidylinositol-4-phosphate 3-kinase catalytic subunit type 2 alpha [Source:HGNC Symbol;Acc:HGNC:8971] | 9301 | -0.256 | -0.2055 | No |
| 38 | IPPK | inositol-pentakisphosphate 2-kinase [Source:HGNC Symbol;Acc:HGNC:14645] | 9547 | -0.278 | -0.2027 | No |
| 39 | PIP5K1A | phosphatidylinositol-4-phosphate 5-kinase type 1 alpha [Source:HGNC Symbol;Acc:HGNC:8994] | 9851 | -0.306 | -0.2021 | No |
| 40 | ITPKA | inositol-trisphosphate 3-kinase A [Source:HGNC Symbol;Acc:HGNC:6178] | 10450 | -0.367 | -0.2196 | Yes |
| 41 | INPP1 | inositol polyphosphate-1-phosphatase [Source:HGNC Symbol;Acc:HGNC:6071] | 10454 | -0.367 | -0.1908 | Yes |
| 42 | PLCG1 | phospholipase C gamma 1 [Source:HGNC Symbol;Acc:HGNC:9065] | 10722 | -0.395 | -0.1804 | Yes |
| 43 | SYNJ1 | synaptojanin 1 [Source:HGNC Symbol;Acc:HGNC:11503] | 11577 | -0.512 | -0.2065 | Yes |
| 44 | IMPA2 | inositol monophosphatase 2 [Source:HGNC Symbol;Acc:HGNC:6051] | 11684 | -0.530 | -0.1729 | Yes |
| 45 | PTEN | phosphatase and tensin homolog [Source:HGNC Symbol;Acc:HGNC:9588] | 11836 | -0.556 | -0.1408 | Yes |
| 46 | INPP5E | inositol polyphosphate-5-phosphatase E [Source:HGNC Symbol;Acc:HGNC:21474] | 11882 | -0.568 | -0.0995 | Yes |
| 47 | INPPL1 | inositol polyphosphate phosphatase like 1 [Source:HGNC Symbol;Acc:HGNC:6080] | 12335 | -0.687 | -0.0804 | Yes |
| 48 | PIKFYVE | "phosphoinositide kinase, FYVE-type zinc finger containing [Source:HGNC Symbol;Acc:HGNC:23785]" | 12347 | -0.690 | -0.0268 | Yes |
| 49 | INPP5B | inositol polyphosphate-5-phosphatase B [Source:HGNC Symbol;Acc:HGNC:6077] | 12723 | -0.884 | 0.0138 | Yes |
Table: GSEA details [plain text format]

  

Fig 2: KEGG\_INOSITOL\_PHOSPHATE\_METABOLISM      
 Blue-Pink O' Gram in the Space of the Analyzed GeneSet

  

Fig 3: KEGG\_INOSITOL\_PHOSPHATE\_METABOLISM: Random ES distribution      
 Gene set null distribution of ES for **KEGG\_INOSITOL\_PHOSPHATE\_METABOLISM**
